# Comparison of two commercial detector arrays for IMRT quality assurance

**DOI:** 10.1120/jacmp.v10i2.2942

**Published:** 2009-04-29

**Authors:** Jonathan G. Li, Guanghua Yan, Chihray Liu

**Affiliations:** ^1^ Department of Radiation Oncology University Of Florida Gainesville FL 32610‐0385; ^2^ Department of Nuclear and Radiological Engineering University of Florida Gainesville FL 32611‐8300 U.S.A.

**Keywords:** intensity modulation, treatment verification, quality assurance, IMRT

## Abstract

Two commercially available detector arrays were compared for their use in the quality assurance of patient‐specific IMRT treatment plans: one a diode‐based array (MapCHECK) and the other an ion chamber‐based array (MatriXX). The dependence of the response of detectors on field size, dose rate, and radiation energy was measured and compared with reference measurements using a Farmer‐type ionization chamber. The linearity of the detector response, short‐term and long‐term reproducibility, statistical uncertainty as a function of delivered dose, and the validity of the array calibration were also examined to understand the stability and uncertainty of the systems. No field size or SSD dependence was observed within the range of the field sizes and SSDs used in the study at both 6 MV and 18 MV photon energies. Both detector arrays showed negligible errors (<1%) when measuring doses of more than ~8 cGy, but exhibited errors of ~3% when measuring doses on the order of 1 cGy. While the MapCHECK showed a stable short‐term reproducibility to within measurement error, the MatriXX showed a slow but continuous increase in readings during the initial one‐hour period (about 0.8%). The MapCHECK also showed a slightly better array sensitivity correction with all the detectors having less than 1% discrepancy and more than 90% of the detectors within 0.5% variation, whereas about 60% of the MatriXX detectors showed a less than 0.5% variation and ~8% exhibited a larger than 1% discrepancy. MatriXX detectors also displayed a volume‐averaging effect consistent with its detector size of ~4.5 mm in diameter. Excellent passing rates were obtained for both detector arrays when compared with the planar dose distributions from the treatment planning system for three 6 MV IMRT fields and three 18 MV IMRT fields after the volume‐averaging effect of the MatriXX was taken into account.

PACS number: 87.55.km; 87.55.Qr; 87.56.Fc

## I. INTRODUCTION

Verification of patient‐specific intensity‐modulated radiation therapy (IMRT) plans using two‐dimensional (2D) detector arrays has become increasingly popular due to their ease of use and immediate readout of the results. After the treatment plan is approved, a phantom plan is generated where each beam is delivered perpendicularly to a fat‐surfaced solid phantom. A planar dose at a certain depth can be extracted from the treatment planning system (TPS) and compared with measurement using 2D detector arrays in the same geometry at the depth of interest. Comparison can be made on a beam‐per‐beam basis or compositely by adding dose from all the beams. 2D dose distribution analysis tools based on the percent dose difference, distance to agreement (DTA), or the Gamma Index, have been developed and implemented commercially.^(^ ^1^ ^–^ ^5^ ^)^ The acquisition of a 2D dose distribution and the real‐time analysis capabilities have made 2D detector arrays preferable to single ion chamber or film measurement for IMRT pre‐treatment verification.

Two types of 2D detector arrays are commercially available with the primary purpose of providing patient‐specific IMRT QA tools: the MapCHECK diode array (Model 1175, Sun Nuclear Corp., Melbourne, FL), and two commercial models of the ionization chamber array (ImRT MatriXX, Scanditronix Wellhofer GmbH, Germany; and seven29, PTW, Freiburg, Germany). Both kinds of detector arrays have been studied in some detail. Jursinic and Nelms^(5)^ and Létourneau et al.^(6)^ examined the linearity and temperature characteristics of the MapCHECK detectors and found that the diode response is linear within the range of the radiation dose delivered (up to 300 cGy). A temperature dependence of about one‐half percent per degree C was also noted. Buonamici et al.^(7)^ compared the MapCHECK and film measurement for IMRT QA and concluded that the diode matrix may effectively replace both film dosimetry and ionimetric measurements in routine IMRT QA. Amerio et al.^(8)^ and Stasi et al.^(9)^ described the design principle and dosimetric properties of a prototype ionization chamber array, which was the basis for the MatriXX. The seven29 has also been described by several authors (Poppe et al.^(10)^ Spezi et al.^(11)^). However, most of the studies were performed with only 6 MV photon beams, and no direct comparison between the two kinds of detector arrays was made under the same conditions.

In general, there are technical concerns over the use of both diode and ion‐chamber arrays for performing QA measurements. The diode array detectors are small (<1 mm), making them ideal for measuring complex IMRT planar dose distributions with minimal volume averaging effect. On the other hand, diodes are known to suffer from radiation damage, energy, field size, and dose‐rate dependencies.^(^ ^12^ ^–^ ^13^ ^)^ Proper evaluations of their response characteristics are essential before their clinical use. Ion chamber‐based detector arrays are known to have insignificant energy and dose‐rate dependence for MV photon beams, but require a larger sensitive volume, with diameters on the order of 5 mm for each chamber, to gain signal and will therefore exhibit a volume averaging effect in steep dose gradient regions.^(14)^ Measurement of dose distributions with steep gradients is necessary in the characterization of IMRT dose distributions. Thus, the effect of volume averaging needs to be carefully characterized and possibly considered in the interpretation of verification results.

In this work, two different detector arrays were evaluated for IMRT pre‐treatment verification: the ion chamber‐based ImRT MatriXX array and the diode‐based MapCHECK 1175 array. Our aim is to study detector‐response dependence on field size, dose rate, radiation energy, and detector size by evaluating their effect on the commonly used percent dose difference and DTA criteria for IMRT planar dose comparison. The linearity of the detector response, short‐term and long‐term reproducibility, statistical uncertainty as a function of delivered dose, and the validity of the array calibration were also examined. The arrays' ability to measure dose distributions with both the 6 MV and 18 MV photon beams were verified by comparing the measurements with planar dose distributions from the TPS.

## II. MATERIALS AND METHODS

The ImRT MatriXX is a pixel ionization chamber array of 1020 detectors on a 32×32 Cartesian grid. The detector spacing is 0.76 cm, covering a total area of $23.6\times 23.6\;{\text{cm}}^{2}$. Each individual ion chamber consists of a vented parallel plate chamber with a diameter of 0.45 cm and a height of 0.5 cm, resulting in a sensitive volume of 0.08 cm^3^. The uniformity correction of the array was determined by the manufacturer and cannot be changed by the user. Following the manufacturer's recommendations, the MatriXX was given a 15‐minute warm‐up time and ≥10 Gy of pre‐irradiation before each use. Additional warm‐up time was given, as discussed later. The MapCHECK contains 445 n‐type solid state diode detectors. The inner 221 detectors cover the central $10\times 10\;{\text{cm}}^{2}$ and are arranged in a zigzag pattern so that the diagonal spacing between detectors is 0.707 cm. The outer 224 detectors are arranged in a similar pattern, but with a diagonal spacing between detectors of 1.414 cm. The array covers an area of $22.0\times 22.0\;{\text{cm}}^{2}$. The active detector area of each diode is $0.8\times 0.8\;{\text{mm}}^{2}$. The relative sensitivity differences between the detectors were obtained through a manufacturer specified procedure.^(15)^ No warm‐up time was given for the MapCHECK. For both devices, the delivery dose was calibrated by delivering a known dose of radiation using a beam with a $10\times 10\;{\text{cm}}^{2}$ field size under reference conditions. All the measurements were done using a dual energy linear accelerator (Synergy, Elekta Oncology Systems Ltd, Crawley, UK) equipped with an 80‐leaf multileaf collimator (MLC) with 1 cm leaf width. The effective depths of the detectors were taken from the manufacturer‐specified values. Additional solid water slabs were added to both arrays to position the detectors at 10 cm of water‐equivalent depth. The radiation beam was perpendicular to the phantom surface and the detector arrays for all measurements.

The linearity of the detectors was assessed by delivering varying amounts of radiation dose with a 6 MV photon beam, from 1 MU to 300 MU (1 MU=0.88 cGy) using a $26\times 26\;{\text{cm}}^{2}$ field size at 100 cm source‐to‐detector distance (SDD). The readings from the central diode of the MapCHECK were correlated with the delivered dose. Since the MatriXX did not have a detector at the central axis, the average of the central four chambers was used for this purpose. The same data sets were also used to study the statistical uncertainty as a function of delivered dose. Using the planar dose distribution measured with 300 MU as baseline, measured dose fluctuations around the baseline can be calculated for smaller delivered MU. For each detector, *i*, a percent error (PE) was calculated as

(1) $$PE_{i}(M)=\left[R_{i}(M)\times \frac{\bar{R}(M=300)}{\bar{R}(M)}-R_{i}(M=300)\right]÷R_{i}(M=300)\times 100\%$$

where $R_{i}(M)$ and $R_{i}(M=300)$ are the readings of the detector *i* that received *M* MU and 300 MU, respectively, and $\bar{R}(M)$ and $\bar{R}(M=300)$ are the average readings of all the detectors that received *M* MU and 300 MU, respectively. The standard deviation of *PE* of all the detectors as a function of *M* was then calculated for the two arrays.

Short‐term and long‐term reproducibility was evaluated by repeating the same measurement every 10 minutes over a one‐hour period and every week over a one‐month period using the above setup, with an additional solid water piece with an embedded Farmer‐type ion chamber at a fixed position below the detector array to monitor the accelerator output. The ratios of the raw readings of the MatriXX and the Farmer‐type chamber (without temperature and pressure corrections) were used directly to assess the MatriXX reproducibility, as both readings were affected in the same way by temperature and pressure variations. On the other hand, the Farmer‐type chamber readings were first corrected for the temperature and pressure before being used to normalize the MapCHECK readings. No temperature correction was attempted for the diode readings, although the temperature variation for all the measurements was small ≤1.2 °C. Both the absolute calibration and array calibration files for the two arrays were kept the same throughout the measurement. Since we were interested in evaluating the reproducibility of all the detectors, the beam tuning of the linac was carefully monitored using the linac's radial and transverse ion chamber plates to make sure beam symmetry was kept consistent to within 0.5% during the long‐term reproducibility measurement.

One practical aspect to note is that since our institution has been using the same MapCHECK device for IMRT patient QA for the past three years, the MapCHECK experienced extensive use before and during the one‐month period, whereas the MatriXX was not in clinical use. Therefore, the effect of the varying amount of radiation to the array sensitivity was not taken into account for the comparison.

The validity of the array calibration was checked using the method as described by Létourneau et al.^(6)^ Each of the arrays was irradiated with an open $26\times 26\;{\text{cm}}^{2}$ 6 MV photon beam at 0° and 180° rotation of the detector array. Since the radiation at the same position was recorded by two different detectors, the subtraction of the two matrices after proper rotation would reveal any array calibration errors. Note that the result of this simple procedure has only statistical meaning, as two detectors at the same position (in the room coordinate) before and after rotation that have the same calibration error would display no error in this test.

The response of the detectors as a function of field size (Scp) and SDD for 6 MV and 18 MV photon beams was studied by first measuring the output factors with field sizes ranging from $4\times 4\;{\text{cm}}^{2}$ to $22\times 22\;{\text{cm}}^{2}$ at 100 SDD and 10 cm depth in a solid water phantom. The results were compared with those obtained using a Farmer chamber measurement in the same geometry. The SDD dependence (or dose rate dependence) was measured by varying the SDD from 80 cm to 150 cm using the same solid water phantom with a fixed field size of $10\times 10\;{\text{cm}}^{2}$ and the results were compared with the Farmer chamber measurement. Only the central diode for the MapCHECK and one of the four chambers near the center for the MatriXX were used for these measurements.

To study the effect of the finite size of the MatriXX detectors on measuring complex IMRT dose distributions, the response functions of a single parallel plate chamber of the MatriXX were determined. A half beam‐blocked $10\times 10\;{\text{cm}}^{2}$ field was created and the MapCHECK and the MatriXX were, in turn, used in single detector mode to measure the beam penumbra at 10 cm depth using 1.0 mm steps, covering±15 mm of the beam edge. Accurate movement of the detectors was achieved using a robotic treatment couch top (HexaPOD, Medical Intelligence, Germany). Profiles perpendicular to the leaf movement direction were obtained with both the 6 MV and 18 MV photon beams, and were normalized to the respective readings at the center of a symmetric $10\times 10\;{\text{cm}}^{2}$ field. The profiles obtained with the MapCHECK diode were assumed to be the true beam edge profiles as the diode size was small (0.8 mm). The response functions of the MatriXX were assumed to be Gaussian and a least‐squares best fit was obtained to determine the widths of the Gaussians. This was accomplished by minimizing the difference between the MatriXX measured profile and the profile obtained by convolving the MapCHECK measured profile with the Gaussians.

To evaluate the ability of the detector arrays in measuring planar dose distributions, three 6 MV IMRT treatment fields and three 18 MV IMRT treatment fields previously used for patient treatment at our institution were selected. The step‐and‐shoot IMRT treatment fields were generated using a commercial TPS (Pinnacle^3^, version 7.6c, Philips Medical Systems, Madison, WI) with direct machine parameter optimization (DMPO) option, which directly optimizes the shape and weight of each MLC segment. Minimum segment area and minimum segment MU were set to 4 cm^2^ and 3 MU, respectively. All six IMRT fields had similar fluence modulations, with the number of segments ranging from 8 to 11. The TPS was commissioned using true beam profiles, free from volume‐averaging effect, and which were extracted from ion chamber‐measured profiles using a recently‐developed methodology.^(16)^ Planar dose distributions at 10 cm depth and with 90 cm SSD in a solid water phantom with gantry angle set to 0° (IEC convention) were measured with both the MapCHECK and MatriXX, and compared with TPS calculated dose distributions under identical geometric setup.

Quantitative comparisons between the MapCHECK and MatriXX measured dose distributions and TPS calculated planar dose distributions were performed using software developed in MATLAB (MathWorks, Inc., Natick, MA). Commonly‐used percent dose difference and DTA criteria of 3%/3 mm and 2%/2 mm were adopted. The algorithm employed to compute the percentage of the points passing the acceptance criteria or passing rate was the same as the one used in the MapCHECK commercial software in the absolute dose comparison mode.^(15)^ A measurement point was included in the analysis if (1) the measured dose was above 10% of the maximum dose of the field, or (2) it was an interior point.^(15)^ No interpolation was applied to the dose distributions measured with the detector arrays. Both the original TPS calculated dose distributions and the TPS dose distributions convolved with a Gaussian function determined from the detector response test were used to compare with the MatriXX measurements.

## III. RESULTS

**Figure 1:**
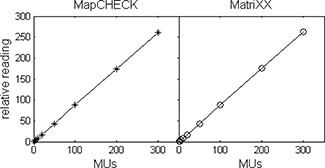
Response linearity of the two detector arrays. The relative reading is plotted as a function of delivered dose from 1 MU to 300 MU (1 MU=0.88 cGy) in a 6 MV photon beam.

**Figure 2:**
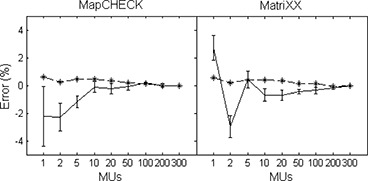
The percent error of the measured output as a function of delivered dose from 1 MU to 300 MU (1 MU=0.88 cGy) using output measured at 300 MU as a reference. The error bars represent the statistical errors for all the detectors as determined using Eq. 1. The dashed lines represent the linac output as monitored using a Farmer chamber.

The response of the detectors as a function of delivered dose is shown in Fig. 1, where the readings from the central diode and the average of the central four chambers were correlated with the delivered dose. Both detectors show a linear response with $R^{2}$ values of better than 0.999. The uncertainty in measuring low dose can be better observed in Fig. 2, where the percent error for the central diode and the average of the central four chambers, $PE_{0}(M)$, as calculated using

(2) $$PE_{0}(M)=\left[R_{0}(M)\times (300/M)-R_{0}(M=300)\right]÷R_{0}(M=300)\times 100\%$$

is plotted against the delivered MU, where $R_{0}(M)$ and $R_{0}(M=300)$ are the detector readings with *M* and 300 MUs, respectively. Also plotted are the standard deviations of the percent error of all the detectors as a function of delivered MU as calculated using (Eq. 1). Both detector arrays showed negligible errors (<1%) when measuring more than 10 MU, corresponding to approximately 8 cGy. The MapCHECK displayed a progressive under response with smaller MUs, whereas the MatriXX did not show any systematic pattern. The linac output for small MUs was accurate to within 0.5%, as monitored with a Farmer chamber.

**Figure 3:**
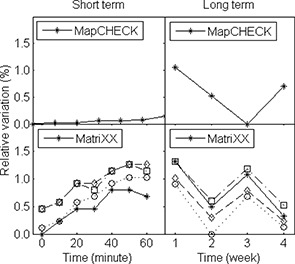
Detector response variation during a period of one hour (short term) and one month (long term). The linac output variation was monitored and corrected using a Farmer chamber. The MatriXX readings were corrected for temperature and pressure variations during the long‐term tests; no correction was applied to the MapCHECK readings.

The short‐term and long‐term reproducibility of the two detector arrays is shown in Fig. 3. The signals from the central diode of the MapCHECK and the central four chambers of the MatriXX were plotted as a function of time during the one‐hour and one‐month period after proper correction of the linac output variation. While the MapCHECK showed a stable short‐term response to within the measurement errors, the MatriXX showed a continuously slow increase in reading during the one‐hour period (about 0.8%). In light of this finding, the MatriXX was given a one‐hour warm‐up time in addition to the manufacturer's recommended warm‐up procedures for all the other measurements. Both detectors showed a fluctuation of about 1% during the one‐month period.

**Figure 4:**
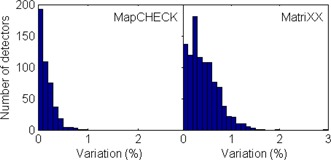
Histogram of the array calibration uncertainties for all the detectors using the rotational method, as described in the text.

The result of the array calibration check is shown in Fig. 4, where the percent difference of the signals acquired at 0^°^ and 180^°^ rotation of the detector array about the beam axis at all the detector positions were histogramed. The MapCHECK showed a slightly better sensitivity correction with all the detectors having less than 1% discrepancy and more than 90% of the detectors within 0.5% variation. On the other hand, about 60% of the MatriXX detectors showed a variation of less than 0.5% and ~8% exhibited a discrepancy larger than 1%, with one outlier at 3%. The array calibration check is especially important for MatriXX, as the calibration file was supplied by the manufacturer and the user does not have the option to recalibrate the array. It is therefore important to check the validity of the array calibration before clinical use and to evaluate the variation of the detector response to determine when it's necessary for recalibration.

**Figure 5:**
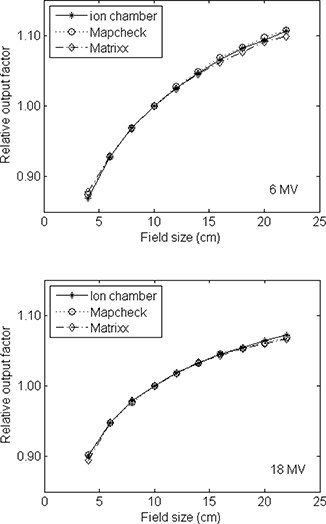
Relative output as a function of field size for the 6 and 18 MV photon beams measured with the MapCHECK and MatriXX, and compared with the measurement using a Farmer‐type ion chamber. Measurement was done in a solid water phantom at 100 cm source to detector distance (SDD) and 10 cm depth. Field sizes varied from $4\times 4\;{\text{cm}}^{2}$ to $22\times 22\;{\text{cm}}^{2}$.

**Figure 6:**
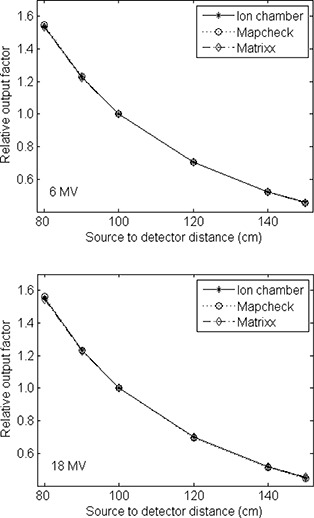
Relative output as a function of SDD for the 6 and 18 MV photon beams measured with the MapCHECK and MatriXX, and compared with the measurement using a Farmer‐type ion chamber. The depth of the detectors was at 10 cm with a fixed field size of $10\times 10\;{\text{cm}}^{2}$. The SDDs varied from 80 cm to 150 cm.

The response of the detectors as a function of field size (Scp) and SDD for 6 MV and 18 MV photon beams are displayed in Figs. 5 and 6, respectively, together with the results using a Farmer chamber in the same geometry. Both the MapCHECK and MatriXX agreed with the Farmer chamber measurement to within 1% for the range of field sizes and SDDs tested for both photon energy beams. The diode result is especially surprising since most diode systems showed significant field‐size and SSD (instantaneous dose‐rate) dependence, and correction factors are necessary if used for in‐vivo Dosimetry.^(12)^

**Figure 7:**
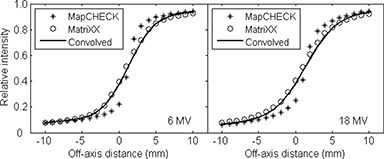
Beam profiles of a half‐beam blocked $10\times 10\;{\text{cm}}^{2}$ field measured with the MapCHECK (^*^) and MatriXX (o) in the single detector mode at 10 cm depth with 1 mm steps, covering ±15 mm of the beam edge. Accurate movement of the detectors was achieved using a robotic treatment couch top. The convolved profiles using the best fit Gaussians are also shown as solid curves with σ of 2.4 mm and 2.6 mm for the 6 and 18 MV beams, respectively.

The beam profiles in the penumbra regions of a half‐beam blocked field as measured by the two detector arrays in the single detector mode are shown in Fig. 7. Also shown are the profiles obtained by convolving the best‐fit Gaussians with the MapCHECK‐measured profiles. The volume averaging of the ion chamber array in the measured beam penumbra is apparent, with larger penumbra measured using the MatriXX. A fit to the MatriXX‐measured profile using the MapCHECK‐measured profile convolved with a Gaussian function resulted in σ of 2.4 mm and 2.6 mm, respectively, for the 6 MV and 18 MV photon beams, corresponding to full width at half maximum (FWHM) of about 5.8 mm. These are very similar to the radius of the MatriXX chambers (2.3 mm) as would be expected from similar investigations.^(14)^

The passing rates between the MapCHECK and TPS calculated dose distributions and between the MatriXX and TPS calculated dose distributions for three 6 MV and three 18 MV IMRT fields are summarized in Table 1. Both the original TPS calculated dose distributions and the TPS calculated dose distributions convolved with a Gaussian with a σ of 2.5 mm were used to compare with the MatriXX measurement. With the 3%/3 mm criteria, all comparisons achieved better than 95% passing rates, with slightly better passing rates for the 6 MV fields than the 18 MV fields. Noticeable improvements in passing rates were obtained when the MatriXX measured dose distributions were compared with the TPS calculated dose distributions after the latter were convolved with a Gaussian function to take into account the volume‐averaging effect of the MatriXX detectors, especially when the 2%/2 mm evaluation criteria were used. Even with the more stringent criteria of 2%/2 mm, good passing rates were obtained for all the comparisons, with the average passing rates of more than 90% for both energies. No significant differences were observed between the two detector arrays.

**Table 1:** Passing rates between MapCHECK and TPS calculated dose distributions and MatriXX and TPS calculated dose distributions for three 6 MV IMRT fields and three 18 MV IMRT fields. Both the original TPS calculated dose distributions and the TPS calculated dose distributions convolved with a Gaussian with a σ of 2.5 mm were used to compare with the MatriXX measured dose distributions.

	*MapCHECK with TPS*		*MatriXX with TPS (Original)*		*MatriXX with TPS (Convolved)*	
	3%/3 mm	2%/2 mm	3%/3 mm	2%/2 mm	3%/3 mm	2%/2 mm
6 MV Field A	98.3%	94.4%	98.1%	93.3%	100.0%	96.1%
6 MV Field B	100.0%	96.8%	97.6%	90.5%	99.2%	94.8%
6 MV Field C	99.6%	96.1%	97.9%	92.7%	98.8%	95.3%
**Average (6 MV)**	**99.3%**	**95.8%**	**97.9%**	**92.2%**	**99.3%**	**95.4%**
18 MV Field A	97.8%	94.9%	96.3%	91.4%	96.9%	94.3%
18 MV Field B	95.3%	93.2%	93.6%	89.8%	95.2%	93.9%
18 MV Field C	99.7%	96.1%	95.8%	90.7%	97.6%	95.1%
**Average (18 MV)**	**97.6%**	**94.7%**	**95.2%**	**90.6%**	**96.6%**	**94.4%**

## IV. DISCUSSION

Our results demonstrate that both detector arrays present the required characteristics for accurate planar dose measurements as required in clinical patient specific IMRT QA. No significant field size or SSD dependence was observed within the range of the field sizes and SSDs employed in the study. Concerns about field size, dose rate, and radiation damage influencing diode detectors do not appear to be warranted given the results of our study. Saini and Zhu^(13)^ found that the buildup material on the top and around the diode die can significantly affect the field size dependence, and can be minimized by suitably chosen buildup material. The minimal dose‐rate dependence of the MapCHECK diodes is, in part, due to the designed increase in recombination‐generation centers that enables the indirect recombination rate to remain nearly constant over the instantaneous dose rate range experienced in radiotherapy applications.^(17)^ Even after 4 years of clinical use in our institution and approximately 300 patient QA measurements per year, the diode array demonstrated excellent performance over a reasonable clinical range of irradiation conditions.

**Figure 8:**
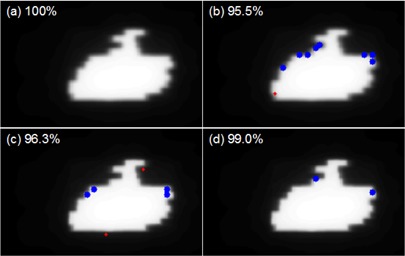
DTA comparison between MatriXX/MapCHECK measurements and TPS calculations of one of the IMRT segments. The TPS calculations were done using both a beam model (BM1) commissioned using a finite‐sized ion chamber (CC04) measured beam profiles and a beam mode (BM2) commissioned using true beam profiles. DTA criteria of 2%/2 mm were used for the comparison and the passing rates are labeled in the top left corners. The blue dots represent failed points where the measurements are lower than TPS calculations; the red dots represent failed points where the measurements are higher than TPS calculations. comparison between MatriXX and BM1; (b) comparison between MapCHECK and BM1; (c) comparison between MatriXX and BM2; (d) comparison between MapCHECK and BM2.

The finite size of the ion‐chamber array resulted in a measurable volume‐averaging effect, with a FWHM of about 5.8 mm, assuming a Gaussian response function. This volume‐averaging effect should be properly taken into account when comparing measured planar dose distributions with those from a TPS for IMRT QA. In this work, this was done by convolving the TPS calculation with a Gaussian function with the experimentally‐determined width before comparing with the MatriXX‐measured dose distributions. This procedure is warranted because our TPS was commissioned using true beam profiles free of volume‐averaging effect.^(16)^ This may not be applicable if beam profiles measured with finite‐sized ion chambers are used directly for beam commissioning. If the size of the ion chamber used to collect beam commissioning data is comparable to the size of the MatriXX detectors, direct comparison between the MatriXX measurement and TPS calculation may produce erroneously high passing rates, as both the measurement and TPS calculation suffer from similar volume‐averaging effect. This would also degrade the passing rates if MapCHECK is used for the measurement. Yan et al.^(16)^ found that significant improvement in passing rates between TPS calculation and MapCHECK measurement could be achieved if the TPS was commissioned using true beam profiles, especially when more stringent criteria such as 2%/2 mm DTA were used. Figure 8 shows a DTA comparison between MatriXX/MapCHECK measurements and TPS calculations of one of the IMRT segments. The TPS calculations were done using both a beam model (BM1) commissioned using a finite‐sized ion chamber measured beam profiles (CC04, Scanditronix Wellhofer, Bartlett, TN) and a beam mode (BM2) commissioned using true beam profiles. When BM1 was used, MatriXX resulted in a higher passing rate than MapCHECK due to similar volume‐averaging effect of BM1 and MatriXX. The use of BM2 improved the passing rate for MapCHECK significantly (Fig. 8(d), while at the same time reduced the passing rate for MatriXX (Fig. 8(c). However, the result is almost identical to Fig. 8(a) if the planar dose distribution from BM2 was first convolved with the Gaussian response function before comparing with the MatriXX measurement. Note that most of the failed points occur in the penumbra area, where the volume‐averaging effect is most pronounced. It is therefore important to understand the volume‐averaging effect of both the TPS and the QA device before meaningful interpretation of IMRT QA results can be made.

While the MapCHECK used a wide‐field calibration technique to obtain the relative sensitivity differences between the detectors, the array calibration for the MatriXX was supplied by the manufacturer. It is important to check the validity of the array calibration before clinical use and to monitor its change over time. One way to check this is to compare the array‐measured beam profiles with scanned beam profiles with ion chambers in water. This procedure is time‐consuming. Létourneau et al.^(6)^ described a rotational method which was used here for array calibration check. We recommend that the manufacturers provide a built‐in method similar to the one used here (Fig. 4) to allow the users to easily check the validity of the array calibration and determine when it's necessary for recalibration. Annual recalibration of the MapCHECK was recommended by the manufacturer.^(15)^ We found no systematic trend for our IMRT QA results after each array calibration and found this recalibration interval to be adequate.

The effective depths of the detectors for both devices were taken from the manufacturer‐specified values and were not verified. This should not be a concern for the current application, since the arrays were calibrated by delivering a known dose of radiation to obtain the raw reading to dose conversion factors in the same geometry. With the increasing interest in rotational or intensity‐modulated arc therapy, both devices have been proposed for use as a pretreatment verification tool. Due to the finite size of the ion chambers in the MatriXX device, the effective point of measurement needs to be determined for such application. Moreover, directional dependence of the detectors becomes important and needs to be carefully evaluated. Recently, Van Esch et al.^(18)^ examined the suitability of an ion‐chamber array (seven29) for pretreatment QA of rotational therapy. They found significant directional dependence, with detector responses of 4% lower for 18 MV and 8% lower for 6 MV photon beams compared with TPS calculation when the array was irradiated from the rear, as compared to agreement within 1.0% when the array was irradiated from the front. The authors proposed a compensation cavity in the phantom, resulting in measurement accuracy comparable to that of single ion chambers. Also, the effective point of measurement was found to be 2.5 mm below the upper electrode when irradiated from the anterior‐posterior direction, resulting in small calibration corrections. Similar studies need to be done for any other devices to ensure the integrity of QA results.

## V. CONCLUSIONS

Two commonly‐used detector arrays, the diode‐based MapCHECK and the ion chamber‐based MatriXX, were examined for their use in the quality assurance of patient‐specific IMRT treatment plans. No field size or SSD dependence was observed within the range of the field sizes and SSDs used in the study at both 6 MV and 18 MV photon energies. Both detector arrays showed negligible errors (<1%) when measuring doses of more than ~8 cGy, but exhibited progressively larger errors when measuring lower doses. While the MapCHECK showed a stable short‐term reproducibility to within measurement error, the MatriXX showed a slow but continuous increase in readings during the initial one‐hour period (about 0.8%). Longer warm‐up times than those recommended by the manufacturer seem necessary in order to achieve more accurate results. The MapCHECK also showed a slightly better array sensitivity correction, with all the detectors having less than 1% discrepancy and more than 90% of the detectors within 0.5% variation, whereas about 60% of the MatriXX detectors showed a less than 0.5% variation and ~8% exhibited a larger than 1% discrepancy. While the volume averaging of the ion‐chamber array was measurable, it can be properly taken into account by convolving the TPS calculation with a Gaussian function before comparing with the MatriXX measurements. Excellent passing rates were obtained for both detector arrays when compared with the planar dose distributions from the treatment planning system for three 6 MV IMRT fields and three 18 MV IMRT fields after the volume‐averaging effect of the MatriXX was taken into account. While we have found excellent performance in both units, institutions employing these devices for patient specific QA should validate the performance of any units before clinical use and establish an ongoing clinical QA program to periodically monitor their performance.

## ACKNOWLEDGEMENTS

The authors would like to thank Scanditronix Wellhofer for the loan of the MatriXX device and Jie Shi and Sanjeev Saini of Sun Nuclear Corp. for useful discussion about MapCHECK analysis software. This work was supported in part by NCI grant R01‐CA‐100636.
